# Diaqua­bis­(perchlorato)(1,10-phenanthroline)copper(II)

**DOI:** 10.1107/S160053681003312X

**Published:** 2010-08-21

**Authors:** Kamel Kaabi, Meher El Glaoui, Matthias Zeller, Cherif Ben Nasr

**Affiliations:** a Laboratoire de Chimie des Matériaux, Faculté des Sciences de Bizerte, 7021 Zarzouna, Tunisia; b Youngstown State University, Department of Chemistry, One University Plaza, Youngstown, Ohio 44555-3663, USA

## Abstract

In the title compound, [Cu(ClO_4_)_2_(C_12_H_8_N_2_)(H_2_O)_2_], the Cu^II^ atom is coordinated in a square-planar fashion by the two N atoms of a chelating 1,10-phenanthroline ligand and by two water mol­ecules *trans* to the N atoms. The coordination sphere of the metal atom is augmented by O atoms of two weakly bonded perchlorate anions, thus yielding a strongly distorted CuN_2_O_4_ octa­hedral environment. The crystal packing is stabilized by O—H⋯O hydrogen bonds between the water mol­ecules and the perchlorate anions. In addition, the organic mol­ecules are associated by π–π stacking inter­actions between symmetry-equivalent anti­parallel non-nitro­gen aromatic rings, with inter­planar distances of 3.543 (2) Å.

## Related literature

For common applications of metal-organic coordination compounds, see: Kubo (1976 ▶); Kobel & Hanack (1986 ▶); Pierpont & Jung (1994 ▶); Huskins & Robson (1990 ▶). For a related structure, see: Kaabi *et al.* (2010 ▶). For π-π inter­actions, see: Janiak (2000 ▶).
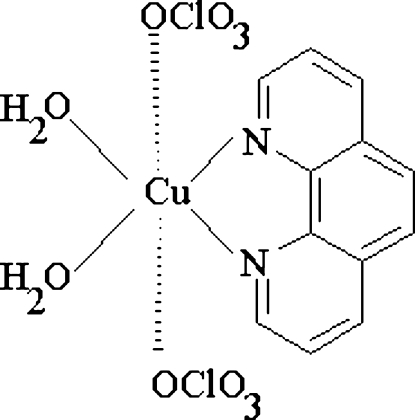


## Experimental

### 

#### Crystal data

[Cu(ClO_4_)_2_(C_12_H_8_N_2_)(H_2_O)_2_]
*M* *_r_* = 478.68
Triclinic, 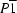

*a* = 7.7882 (10) Å
*b* = 10.4226 (14) Å
*c* = 10.8065 (14) Å
α = 76.567 (1)°
β = 75.227 (2)°
γ = 79.801 (2)°
*V* = 818.50 (19) Å^3^
*Z* = 2
Mo *K*α radiation
μ = 1.72 mm^−1^
*T* = 100 K
0.45 × 0.25 × 0.21 mm

#### Data collection

Bruker APEXII CCD diffractometer
Absorption correction: multi-scan (*SADABS*; Bruker, 2009 ▶) *T*_min_ = 0.587, *T*_max_ = 0.746
17211 measured reflections
4872 independent reflections
4647 reflections with *I* > 2σ(*I*)
*R*_int_ = 0.020

#### Refinement

*R*[*F* ^2^ > 2σ(*F* ^2^)] = 0.024
*wR*(*F* ^2^) = 0.074
*S* = 1.13
4872 reflections
256 parameters
4 restraints
H atoms treated by a mixture of independent and constrained refinement
Δρ_max_ = 0.48 e Å^−3^
Δρ_min_ = −0.53 e Å^−3^

### 

Data collection: *APEX2* (Bruker, 2009 ▶); cell refinement: *SAINT* (Bruker, 2009 ▶); data reduction: *SAINT*; program(s) used to solve structure: *SHELXTL* (Sheldrick, 2008 ▶); program(s) used to refine structure: *SHELXTL*; molecular graphics: *SHELXTL* and *DIAMOND* (Brandenburg, 1998 ▶); software used to prepare material for publication: *SHELXTL*.

## Supplementary Material

Crystal structure: contains datablocks global, I. DOI: 10.1107/S160053681003312X/wm2390sup1.cif

Structure factors: contains datablocks I. DOI: 10.1107/S160053681003312X/wm2390Isup2.hkl

Additional supplementary materials:  crystallographic information; 3D view; checkCIF report

## Figures and Tables

**Table 1:** Selected bond lengths (Å)

Cu1—O2	1.9600 (11)
Cu1—N2	1.9764 (12)
Cu1—O1	1.9784 (10)
Cu1—N1	1.9953 (12)
Cu1—O9	2.3805 (11)
Cu1—O3	2.5508 (11)

**Table 2:** Hydrogen-bond geometry (Å, °)

*D*—H⋯*A*	*D*—H	H⋯*A*	*D*⋯*A*	*D*—H⋯*A*
O1—H1*A*⋯O5^i^	0.82 (2)	1.96 (2)	2.7619 (15)	166 (2)
O1—H1*B*⋯O7^ii^	0.80 (2)	1.95 (2)	2.7486 (16)	175 (2)
O2—H2*A*⋯O5	0.83 (2)	1.95 (2)	2.7566 (16)	164 (2)
O2—H2*B*⋯O8	0.80 (2)	2.20 (2)	2.8339 (18)	136 (2)
O2—H2*B*⋯O8^iii^	0.80 (2)	2.29 (2)	2.8802 (17)	132 (2)

Symmetry codes: (i) 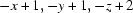; (ii) 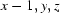; (iii) 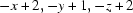.

**Fig. 1.**
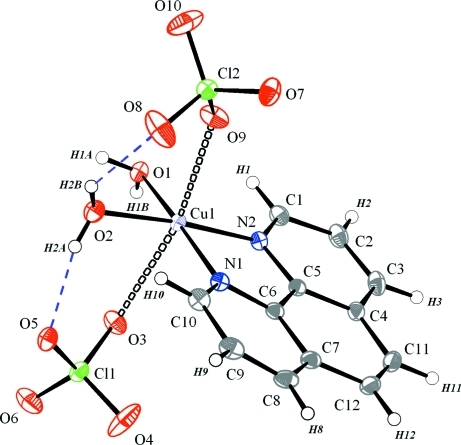
A view of the asymmetric unit of the title compound, showing 50% probability displacement ellipsoids, arbitrary spheres for the H atoms, and the atom numbering scheme.

**Fig. 2.**
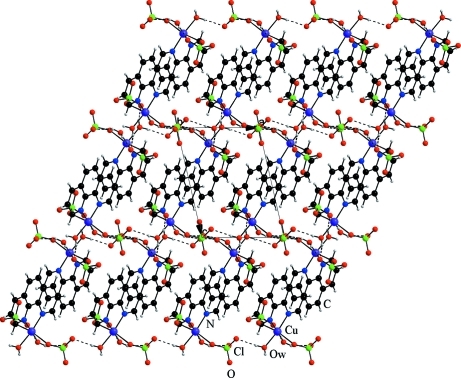
The packing of [Cu(H2O)2(C12H8N2)](ClO4)2, viewed down the b axis. Hydrogen bonds are denoted by dotted lines.

**Fig. 3.**
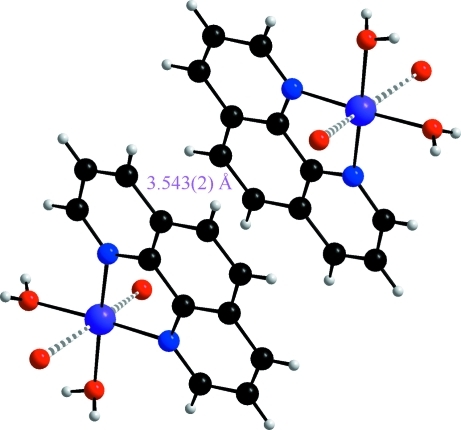
π-π stacking interactions in [Cu(H2O)2(C12H8N2)](ClO4)2.

## supplementary crystallographic information

### Comment

In recent years, there has been an active and concerted attention in developing metal-organic coordination materials owing to their potentially useful magnetic, electric, photochemical and catalytic properties (Kubo, 1976; Kobel & Hanack, 1986; Pierpont & Jung, 1994; Huskins & Robson, 1990). Here, the title compound, [Cu(H_2_O)_2_(C_12_H_8_N_2_)](ClO_4_)_2_, is presented, which has been obtained in continuation of our studies of new coordination compounds (Kaabi, *et al.*, 2010).

In the atomic arrangement of the title material, the Cu ion is surrounded by one 1,10-phenanthroline ligand, two ClO_4_^-^ anions and two water molecules. The Cu^II^ atom is coordinated in a square-planar fashion by two nitrogen atoms of a chelating *o*-phenanthroline ligand and two water molecules *trans* to the nitrogen atoms with Cu—N1 and Cu—N2 distances of 1.9953 (12) and 1.9764 (12) Å, and Cu—O1 and Cu—O2 distances of 1.9784 (10) and 1.9600 (11) Å, respectively. The coordination sphere around the metal is augmented by oxygen atoms of two weakly bound perchlorate anions, thus yielding a strongly distorted octahedral environment for the copper(II) ion (Fig. 1). The two Cu—O distances to the perchlorate anions, 2.3805 (11) and 2.5508 (11) Å, are much longer than the Cu—O(water) distances (Table 1), and especially the longer of these two distances should be regarded as a very weak interaction rather than an actual metal-oxygen bond.

The complexes are interconnected by a set of O—H···O hydrogen bonds between the water molecules and the perchlorate anions (Table 2), leading to the formation of a three dimensional network (Fig. 2). As expected, the ClO_4_^-^ anion has a characteristic tetrahedral geometry where the Cl—O bond lengths and O—Cl—O angles are slightly different from one another and vary with the coordination of the individual O atoms, such as hydrogen bonds or metal coordination. In the title compound, the Cl—O bond lengths vary between 1.4272 (13) and 1.4728 (11) Å and 1.4230 (14) and 1.4490 (11) Å for the two ClO_4_^-^ anions. The O—Cl—O angles range from 107.95 (7) to 111.67 (10)° for the first anion and from 108.62 (7) to 110.48 (11)° for the second anion.

Intermolecular π–π stacking interactions between neighboring non-nitrogen aromatic rings of 1,10-phenanthroline are also observed, with a face-to-face distance of 3.543 (2) Å (Fig. 3), less than 3.8 Å, the maximum value regarded as relevant for π–π interactions (Janiak, 2000).

### Experimental

An aqueous solution of Cu(ClO_4_)_2_ (1 mmol, 0.263 g) was added dropwise to a solution of 1,10-phenanthroline (1 mmol, 0.180 g) in ethanol. The resultant mixture was evaporated at room temperature. Crystals of the title compound, which remained stable under normal conditions of temperature and humidity, were isolated after several days and subjected to X-ray diffraction analysis (yield 63%).

### Refinement

C—H hydrogen atoms were placed in calculated positions with C—H distances in the range 0.93–0.97 Å. The water hydrogen atom postitions were refined with O—H distance restraints of 0.84 (2) Å. The *U*_iso_(H) values of all H atoms were constrained to 1.2 or 1.5× *U*_eq_ of the respective parent atom.

### Crystal data

**Table:**

[Cu(ClO_4_)_2_(C_12_H_8_N_2_)(H_2_O)_2_]	*Z* = 2
*M**_r_* = 478.68	*F*(000) = 482
Triclinic, *P*1	*D*_x_ = 1.942 Mg m^−^^3^
Hall symbol: -P 1	Mo *K*α radiation, λ = 0.71073 Å
*a* = 7.7882 (10) Å	Cell parameters from 6809 reflections
*b* = 10.4226 (14) Å	θ = 2.5–31.1°
*c* = 10.8065 (14) Å	µ = 1.72 mm^−^^1^
α = 76.567 (1)°	*T* = 100 K
β = 75.227 (2)°	Rod, blue
γ = 79.801 (2)°	0.45 × 0.25 × 0.21 mm
*V* = 818.50 (19) Å^3^	

### Data collection

**Table:**

Bruker APEXII CCD diffractometer	4872 independent reflections
Radiation source: fine-focus sealed tube	4647 reflections with *I* > 2σ(*I*)
graphite	*R*_int_ = 0.020
ω scans	θ_max_ = 31.3°, θ_min_ = 2.0°
Absorption correction: multi-scan (*SADABS*; Bruker, 2009)	*h* = −11→11
*T*_min_ = 0.587, *T*_max_ = 0.746	*k* = −14→14
17211 measured reflections	*l* = −15→15

### Refinement

**Table:**

Refinement on *F*^2^	Primary atom site location: structure-invariant direct methods
Least-squares matrix: full	Secondary atom site location: difference Fourier map
*R*[*F*^2^ > 2σ(*F*^2^)] = 0.024	Hydrogen site location: inferred from neighbouring sites
*wR*(*F*^2^) = 0.074	H atoms treated by a mixture of independent and constrained refinement
*S* = 1.13	*w* = 1/[σ^2^(*F*_o_^2^) + (0.0396*P*)^2^ + 0.4561*P*] where *P* = (*F*_o_^2^ + 2*F*_c_^2^)/3
4872 reflections	(Δ/σ)_max_ = 0.003
256 parameters	Δρ_max_ = 0.48 e Å^−^^3^
4 restraints	Δρ_min_ = −0.53 e Å^−^^3^

### Special details

**Table:**

Geometry. All e.s.d.'s (except the e.s.d. in the dihedral angle between two l.s. planes) are estimated using the full covariance matrix. The cell e.s.d.'s are taken into account individually in the estimation of e.s.d.'s in distances, angles and torsion angles; correlations between e.s.d.'s in cell parameters are only used when they are defined by crystal symmetry. An approximate (isotropic) treatment of cell e.s.d.'s is used for estimating e.s.d.'s involving l.s. planes.
Refinement. Refinement of *F*^2^ against ALL reflections. The weighted *R*-factor *wR* and goodness of fit *S* are based on *F*^2^, conventional *R*-factors *R* are based on *F*, with *F* set to zero for negative *F*^2^. The threshold expression of *F*^2^ > σ(*F*^2^) is used only for calculating *R*-factors(gt) *etc*. and is not relevant to the choice of reflections for refinement. *R*-factors based on *F*^2^ are statistically about twice as large as those based on *F*, and *R*- factors based on ALL data will be even larger.

### Fractional atomic coordinates and isotropic or equivalent isotropic displacement parameters (Å^2^)

**Table:**

	*x*	*y*	*z*	*U*_iso_*/*U*_eq_	
C1	0.48090 (19)	1.03799 (14)	0.78959 (15)	0.0155 (2)	
H1	0.4156	1.0302	0.8776	0.019*	
C2	0.4523 (2)	1.15657 (14)	0.70023 (16)	0.0186 (3)	
H2	0.3690	1.2282	0.7281	0.022*	
C3	0.5448 (2)	1.16911 (14)	0.57240 (16)	0.0189 (3)	
H3	0.5258	1.2493	0.5116	0.023*	
C4	0.66839 (19)	1.06189 (14)	0.53204 (14)	0.0151 (2)	
C5	0.69072 (18)	0.94807 (13)	0.62763 (13)	0.0122 (2)	
C6	0.81967 (18)	0.83761 (13)	0.59601 (13)	0.0129 (2)	
C7	0.91968 (19)	0.84133 (15)	0.46757 (14)	0.0160 (3)	
C8	1.0478 (2)	0.72975 (16)	0.44474 (15)	0.0191 (3)	
H8	1.1164	0.7254	0.3591	0.023*	
C9	1.0719 (2)	0.62824 (16)	0.54732 (16)	0.0200 (3)	
H9	1.1602	0.5543	0.5335	0.024*	
C10	0.96612 (19)	0.63344 (15)	0.67308 (15)	0.0174 (3)	
H10	0.9845	0.5623	0.7433	0.021*	
C11	0.7716 (2)	1.06372 (16)	0.40131 (15)	0.0188 (3)	
H11	0.7564	1.1404	0.3356	0.023*	
C12	0.8908 (2)	0.95768 (16)	0.36975 (15)	0.0192 (3)	
H12	0.9557	0.9605	0.2821	0.023*	
Cl1	0.49764 (5)	0.52161 (3)	0.72929 (3)	0.01545 (7)	
Cl2	0.99352 (4)	0.77378 (3)	1.00887 (3)	0.01565 (7)	
Cu1	0.65728 (2)	0.758637 (15)	0.858913 (15)	0.01130 (6)	
N1	0.84121 (16)	0.73496 (12)	0.69669 (12)	0.0131 (2)	
N2	0.59768 (15)	0.93583 (11)	0.75385 (12)	0.0124 (2)	
O1	0.45249 (14)	0.78997 (10)	1.00501 (10)	0.01413 (19)	
H1A	0.446 (3)	0.7260 (18)	1.0657 (18)	0.021*	
H1B	0.357 (2)	0.808 (2)	0.986 (2)	0.021*	
O2	0.70251 (15)	0.57653 (11)	0.95518 (11)	0.0177 (2)	
H2A	0.689 (3)	0.520 (2)	0.918 (2)	0.027*	
H2B	0.786 (3)	0.555 (2)	0.989 (2)	0.027*	
O3	0.45492 (15)	0.64314 (11)	0.77965 (12)	0.0194 (2)	
O4	0.5846 (2)	0.54920 (15)	0.59434 (12)	0.0395 (4)	
O5	0.62326 (14)	0.42954 (10)	0.79995 (11)	0.0176 (2)	
O6	0.33842 (17)	0.46231 (12)	0.74967 (14)	0.0276 (3)	
O7	1.13473 (15)	0.84737 (12)	0.92507 (12)	0.0240 (2)	
O8	1.02607 (18)	0.64014 (13)	0.98634 (18)	0.0372 (4)	
O9	0.82443 (14)	0.83601 (12)	0.97691 (13)	0.0219 (2)	
O10	0.9878 (2)	0.77359 (19)	1.14159 (13)	0.0417 (4)	

### Atomic displacement parameters (Å^2^)

**Table:**

	*U*^11^	*U*^22^	*U*^33^	*U*^12^	*U*^13^	*U*^23^
C1	0.0146 (6)	0.0133 (6)	0.0188 (6)	−0.0006 (5)	−0.0038 (5)	−0.0044 (5)
C2	0.0178 (7)	0.0118 (6)	0.0260 (7)	−0.0001 (5)	−0.0062 (5)	−0.0031 (5)
C3	0.0184 (7)	0.0125 (6)	0.0253 (7)	−0.0030 (5)	−0.0088 (5)	0.0021 (5)
C4	0.0162 (6)	0.0150 (6)	0.0155 (6)	−0.0056 (5)	−0.0064 (5)	0.0004 (5)
C5	0.0123 (6)	0.0128 (5)	0.0127 (6)	−0.0030 (4)	−0.0042 (4)	−0.0020 (4)
C6	0.0115 (6)	0.0146 (6)	0.0137 (6)	−0.0030 (4)	−0.0032 (4)	−0.0039 (5)
C7	0.0143 (6)	0.0210 (6)	0.0146 (6)	−0.0059 (5)	−0.0023 (5)	−0.0055 (5)
C8	0.0155 (6)	0.0273 (7)	0.0167 (7)	−0.0045 (5)	0.0004 (5)	−0.0117 (6)
C9	0.0149 (6)	0.0225 (7)	0.0233 (7)	0.0009 (5)	−0.0013 (5)	−0.0117 (6)
C10	0.0138 (6)	0.0165 (6)	0.0212 (7)	0.0015 (5)	−0.0031 (5)	−0.0058 (5)
C11	0.0207 (7)	0.0208 (7)	0.0153 (6)	−0.0085 (5)	−0.0058 (5)	0.0020 (5)
C12	0.0201 (7)	0.0265 (7)	0.0126 (6)	−0.0097 (6)	−0.0029 (5)	−0.0025 (5)
Cl1	0.01850 (16)	0.01185 (14)	0.01345 (15)	0.00302 (11)	−0.00298 (11)	−0.00183 (11)
Cl2	0.01062 (14)	0.01961 (15)	0.01699 (16)	−0.00126 (11)	−0.00489 (11)	−0.00257 (12)
Cu1	0.01059 (9)	0.01082 (8)	0.01119 (9)	0.00001 (6)	−0.00176 (6)	−0.00135 (6)
N1	0.0114 (5)	0.0138 (5)	0.0140 (5)	−0.0007 (4)	−0.0025 (4)	−0.0034 (4)
N2	0.0107 (5)	0.0117 (5)	0.0144 (5)	−0.0011 (4)	−0.0022 (4)	−0.0026 (4)
O1	0.0117 (4)	0.0152 (5)	0.0138 (5)	−0.0007 (4)	−0.0025 (3)	−0.0006 (4)
O2	0.0173 (5)	0.0162 (5)	0.0179 (5)	0.0019 (4)	−0.0068 (4)	0.0002 (4)
O3	0.0171 (5)	0.0128 (5)	0.0294 (6)	0.0025 (4)	−0.0069 (4)	−0.0076 (4)
O4	0.0498 (9)	0.0364 (7)	0.0130 (6)	0.0210 (6)	0.0030 (5)	0.0024 (5)
O5	0.0173 (5)	0.0136 (4)	0.0185 (5)	0.0028 (4)	−0.0046 (4)	0.0009 (4)
O6	0.0281 (6)	0.0185 (5)	0.0433 (8)	−0.0014 (5)	−0.0200 (5)	−0.0080 (5)
O7	0.0150 (5)	0.0291 (6)	0.0238 (6)	−0.0059 (4)	−0.0039 (4)	0.0046 (5)
O8	0.0221 (6)	0.0175 (6)	0.0769 (11)	0.0048 (5)	−0.0222 (7)	−0.0121 (6)
O9	0.0120 (5)	0.0236 (5)	0.0347 (6)	0.0050 (4)	−0.0109 (4)	−0.0142 (5)
O10	0.0322 (7)	0.0777 (12)	0.0163 (6)	−0.0136 (7)	−0.0070 (5)	−0.0046 (7)

### Geometric parameters (Å, °)

**Table:**

C1—N2	1.3338 (17)	C11—C12	1.360 (2)
C1—C2	1.403 (2)	C11—H11	0.9500
C1—H1	0.9500	C12—H12	0.9500
C2—C3	1.373 (2)	Cl1—O6	1.4272 (13)
C2—H2	0.9500	Cl1—O4	1.4277 (13)
C3—C4	1.413 (2)	Cl1—O3	1.4456 (11)
C3—H3	0.9500	Cl1—O5	1.4728 (11)
C4—C5	1.3978 (19)	Cl2—O10	1.4230 (14)
C4—C11	1.434 (2)	Cl2—O8	1.4369 (13)
C5—N2	1.3600 (18)	Cl2—O7	1.4443 (12)
C5—C6	1.4315 (19)	Cl2—O9	1.4490 (11)
C6—N1	1.3583 (18)	Cu1—O2	1.9600 (11)
C6—C7	1.4033 (19)	Cu1—N2	1.9764 (12)
C7—C8	1.416 (2)	Cu1—O1	1.9784 (10)
C7—C12	1.438 (2)	Cu1—N1	1.9953 (12)
C8—C9	1.369 (2)	Cu1—O9	2.3805 (11)
C8—H8	0.9500	Cu1—O3	2.5508 (11)
C9—C10	1.406 (2)	O1—H1A	0.818 (15)
C9—H9	0.9500	O1—H1B	0.800 (16)
C10—N1	1.3287 (18)	O2—H2A	0.828 (16)
C10—H10	0.9500	O2—H2B	0.796 (16)
			
N2—C1—C2	121.54 (14)	O6—Cl1—O5	109.27 (7)
N2—C1—H1	119.2	O4—Cl1—O5	107.95 (7)
C2—C1—H1	119.2	O3—Cl1—O5	108.49 (7)
C3—C2—C1	119.98 (14)	O10—Cl2—O8	110.48 (11)
C3—C2—H2	120.0	O10—Cl2—O7	109.54 (9)
C1—C2—H2	120.0	O8—Cl2—O7	109.21 (9)
C2—C3—C4	119.50 (13)	O10—Cl2—O9	109.96 (9)
C2—C3—H3	120.2	O8—Cl2—O9	108.62 (7)
C4—C3—H3	120.2	O7—Cl2—O9	109.00 (7)
C5—C4—C3	116.85 (13)	O2—Cu1—N2	174.66 (5)
C5—C4—C11	118.86 (14)	O2—Cu1—O1	88.24 (5)
C3—C4—C11	124.28 (14)	N2—Cu1—O1	91.93 (5)
N2—C5—C4	123.37 (13)	O2—Cu1—N1	96.05 (5)
N2—C5—C6	116.35 (12)	N2—Cu1—N1	83.24 (5)
C4—C5—C6	120.26 (13)	O1—Cu1—N1	172.57 (5)
N1—C6—C7	123.64 (13)	O2—Cu1—O9	90.50 (5)
N1—C6—C5	116.32 (12)	N2—Cu1—O9	94.84 (5)
C7—C6—C5	120.02 (13)	O1—Cu1—O9	84.57 (4)
C6—C7—C8	116.66 (14)	N1—Cu1—O9	101.41 (5)
C6—C7—C12	118.86 (14)	O2—Cu1—O3	79.80 (4)
C8—C7—C12	124.45 (14)	N2—Cu1—O3	94.87 (4)
C9—C8—C7	119.34 (14)	O1—Cu1—O3	87.45 (4)
C9—C8—H8	120.3	N1—Cu1—O3	87.35 (4)
C7—C8—H8	120.3	O9—Cu1—O3	167.63 (4)
C8—C9—C10	120.01 (14)	C10—N1—C6	118.35 (13)
C8—C9—H9	120.0	C10—N1—Cu1	130.12 (11)
C10—C9—H9	120.0	C6—N1—Cu1	111.33 (9)
N1—C10—C9	121.96 (14)	C1—N2—C5	118.73 (12)
N1—C10—H10	119.0	C1—N2—Cu1	129.24 (10)
C9—C10—H10	119.0	C5—N2—Cu1	112.01 (9)
C12—C11—C4	121.18 (14)	Cu1—O1—H1A	111.7 (15)
C12—C11—H11	119.4	Cu1—O1—H1B	114.7 (16)
C4—C11—H11	119.4	H1A—O1—H1B	108 (2)
C11—C12—C7	120.77 (14)	Cu1—O2—H2A	113.0 (16)
C11—C12—H12	119.6	Cu1—O2—H2B	120.8 (17)
C7—C12—H12	119.6	H2A—O2—H2B	112 (2)
O6—Cl1—O4	111.67 (10)	Cl1—O3—Cu1	128.58 (6)
O6—Cl1—O3	109.72 (7)	Cl2—O9—Cu1	127.20 (7)
O4—Cl1—O3	109.67 (8)		
			
N2—C1—C2—C3	0.4 (2)	O2—Cu1—N1—C6	166.74 (9)
C1—C2—C3—C4	0.0 (2)	N2—Cu1—N1—C6	−7.93 (9)
C2—C3—C4—C5	−0.9 (2)	O9—Cu1—N1—C6	−101.53 (9)
C2—C3—C4—C11	179.75 (14)	O3—Cu1—N1—C6	87.29 (9)
C3—C4—C5—N2	1.5 (2)	C2—C1—N2—C5	0.1 (2)
C11—C4—C5—N2	−179.12 (12)	C2—C1—N2—Cu1	−178.36 (11)
C3—C4—C5—C6	−176.98 (12)	C4—C5—N2—C1	−1.1 (2)
C11—C4—C5—C6	2.4 (2)	C6—C5—N2—C1	177.41 (12)
N2—C5—C6—N1	−2.95 (18)	C4—C5—N2—Cu1	177.62 (11)
C4—C5—C6—N1	175.62 (12)	C6—C5—N2—Cu1	−3.86 (15)
N2—C5—C6—C7	179.11 (12)	O1—Cu1—N2—C1	10.63 (13)
C4—C5—C6—C7	−2.3 (2)	N1—Cu1—N2—C1	−175.03 (13)
N1—C6—C7—C8	0.6 (2)	O9—Cu1—N2—C1	−74.08 (13)
C5—C6—C7—C8	178.41 (12)	O3—Cu1—N2—C1	98.24 (12)
N1—C6—C7—C12	−177.39 (13)	O1—Cu1—N2—C5	−167.92 (9)
C5—C6—C7—C12	0.4 (2)	N1—Cu1—N2—C5	6.42 (9)
C6—C7—C8—C9	−2.3 (2)	O9—Cu1—N2—C5	107.37 (9)
C12—C7—C8—C9	175.64 (14)	O3—Cu1—N2—C5	−80.32 (9)
C7—C8—C9—C10	2.0 (2)	O6—Cl1—O3—Cu1	154.80 (8)
C8—C9—C10—N1	0.0 (2)	O4—Cl1—O3—Cu1	−82.19 (11)
C5—C4—C11—C12	−0.6 (2)	O5—Cl1—O3—Cu1	35.50 (10)
C3—C4—C11—C12	178.75 (14)	O2—Cu1—O3—Cl1	−49.19 (9)
C4—C11—C12—C7	−1.4 (2)	N2—Cu1—O3—Cl1	130.42 (9)
C6—C7—C12—C11	1.4 (2)	O1—Cu1—O3—Cl1	−137.86 (9)
C8—C7—C12—C11	−176.41 (14)	N1—Cu1—O3—Cl1	47.44 (9)
C9—C10—N1—C6	−1.7 (2)	O9—Cu1—O3—Cl1	−88.04 (19)
C9—C10—N1—Cu1	172.69 (11)	O10—Cl2—O9—Cu1	−134.98 (11)
C7—C6—N1—C10	1.3 (2)	O8—Cl2—O9—Cu1	−13.97 (13)
C5—C6—N1—C10	−176.52 (12)	O7—Cl2—O9—Cu1	104.93 (10)
C7—C6—N1—Cu1	−174.04 (11)	O2—Cu1—O9—Cl2	42.89 (10)
C5—C6—N1—Cu1	8.10 (14)	N2—Cu1—O9—Cl2	−137.45 (10)
O2—Cu1—N1—C10	−7.94 (14)	O1—Cu1—O9—Cl2	131.07 (10)
N2—Cu1—N1—C10	177.39 (13)	N1—Cu1—O9—Cl2	−53.38 (10)
O9—Cu1—N1—C10	83.79 (13)	O3—Cu1—O9—Cl2	81.01 (19)
O3—Cu1—N1—C10	−87.39 (13)		

### Hydrogen-bond geometry (Å, °)

**Table:**

*D*—H···*A*	*D*—H	H···*A*	*D*···*A*	*D*—H···*A*
O1—H1A···O5^i^	0.82 (2)	1.96 (2)	2.7619 (15)	166 (2)
O1—H1B···O7^ii^	0.80 (2)	1.95 (2)	2.7486 (16)	175 (2)
O2—H2A···O5	0.83 (2)	1.95 (2)	2.7566 (16)	164 (2)
O2—H2B···O8	0.80 (2)	2.20 (2)	2.8339 (18)	136 (2)
O2—H2B···O8^iii^	0.80 (2)	2.29 (2)	2.8802 (17)	132 (2)

Symmetry codes: (i) −*x*+1, −*y*+1, −*z*+2; (ii) *x*−1, *y*, *z*; (iii) −*x*+2, −*y*+1, −*z*+2.
